# Total Replacement of Fishmeal with Plant Sources in European Seabass (*Dicentrarchus labrax*): Growth Parameters, Nutritional Efficiency, Intestinal Health and Economic Profitability

**DOI:** 10.3390/ani16203172

**Published:** 2026-10-09

**Authors:** Edilson Ronny Cusiyunca-Phoco, Zaida Soler-Luque, D. S. Peñaranda, A. Tomás-Vidal, S. Martínez-Llorens, M. Jover-Cerdá

**Affiliations:** 1 Aquaculture and Biodiversity Research Group, Institute for Science and Animal Technology (ICTA), Universitat Politècnica de València, 46022 Valencia, Spain; dasncpea@upv.es (D.S.P.); atomasv@dca.upv.es (A.T.-V.); silmarll@dca.upv.es (S.M.-L.); mjover@dca.upv.es (M.J.-C.); 2 Faculty of Fisheries Engineering, José Faustino Sánchez Carrión National University, Huacho 15136, Peru; 3 School of Agricultural Engineering and Environmental Sciences (ETSIAMN), Universitat Politècnica de València, 46022 Valencia, Spain; zaida.soler@vhir.org

**Keywords:** vegetable raw materials, seabass, intestinal health, intestinal gene expression

## Abstract

The objective of this research was to evaluate the effects of different dietary fishmeal levels (30%, 15% and 0%) on growth, biometric parameters, nutritional efficiency, and intestinal health in juvenile European seabass. The replacement of fishmeal was made with a mixture of vegetable protein sources (soybean meal, wheat gluten, and wheat meal), and synthetic amino acids in isoproteic diets. The experimental period lasted 196 days, and the fish were fed the formulated diets twice a day, with an initial average weight of 40 g/fish. The results showed that it is possible to partially replace fishmeal with an inclusion level of 15% without compromising growth performance. However, the complete substitution of fishmeal resulted in increased viscerosomatic index (VSI) and mesenteric fat (MF), which was also associated with a pro-inflammatory response and moderate immunosuppression at the intestine level. Fish fed the 0 FM diet exhibited reduced growth, poor productive performance, and the lowest survival rates. Furthermore, the expression levels of *Muc2* and *β-actin* genes, together with trends observed for *TGF-β* and *IL-8*, indicated impaired intestinal health and a pronounced immunosuppressive condition in these fish. Economic profitability was found to depend on the market prices of fishmeal and wheat gluten.

## Simple Summary

In the context of sustainable and economically viable aquafeed production, optimizing seabass nutrition by reducing the proportion of fishmeal constitutes a pivotal strategy. The incorporation of a combination of vegetable protein sources, including wheat gluten, wheat meal, and soybean meal, along with Arg, Lys, Met, and Tau, resulted in a fishmeal inclusion level of 15%, without compromising the growth performance and health of the fish.

## 1. Introduction

A key challenge in aquaculture production is the scarcity of animal-derived aquatic protein sources, such as fishmeal (FM), and the associated high cost, which has prompted a search for novel alternative protein sources. This has resulted in an escalating utilization of plant-based protein sources in aquaculture feed formulations. In order to meet the increasing demand and a rapidly expanding population projected to reach 10.3 billion by the 2080s, aquaculture is expected to play a crucial role in supplying fish for responsible human consumption as a sustainable source of nutritious food, and this role is anticipated to continue into the foreseeable future [1,2]. Therefore, aquaculture and the fishing industry play a fundamental role in achieving the Sustainable Development Goals (SDGs) by balancing sound, sustainable management practices with the provision of high-quality food and economic, environmental, and social benefits [3].

In the context of sustainable aquaculture, it is imperative to identify alternative protein sources that reduce reliance on fishmeal in aquaculture feed without causing adverse environmental consequences, despite their limited availability and higher costs. Consequently, the most promising sources are plant- and animal-based proteins [4,5]. These protein sources are incorporated either as individual components or in a mixture, with the objective of reducing the utilization of fishmeal. This is because they share similar amino acid profiles and are free of antinutritional factors (after pretreatment). Moreover, they enhance palatability without compromising production performance, nutrient absorption, metabolism, or intestinal health in fish [4,6,7,8,9,10].

In recent decades, there has been an increasing tendency to utilize combinations of animal and plant protein sources in the formulation of feed for carnivorous fish. This involves a higher proportion of animal proteins and a lower percentage of plant sources. The objective of this approach is to enhance palatability, intestinal health, and feed processing quality. Therefore, it is anticipated that this will improve fish performance and reduce mortality. Meanwhile, some studies have focused on the use of poultry meal, insect meal, soybean meal, and wheat gluten [3,4,5,11,12]. However, in the case of European seabass, a carnivorous species, it is essential to consider not only the attainment of enhanced final weight gain, but also the assessment of intestinal health and integrity, along with the expression of genes associated with a pro-inflammatory response due to the occurrence of poor nutrient absorption, a consequence of an imbalance in the proportion of ingredients incorporated into the diet [13,14,15,16]. Furthermore, the hindgut represents one of the most immunologically relevant intestinal regions in both mammals and several fish species [14,17,18,19,20]. In contrast, in other fish, the locations of intestinal regions or segments, such as the anterior intestine (AI), middle intestine (MI), and posterior intestine (PI), demonstrate that optimal antigen responses vary. Such responses have been reported in European seabass [20], and in the posterior region of the Atlantic cod rectum [21].

Regarding the sustainable feeding of European seabass, it has been demonstrated that 25–50% of fishmeal can be replaced with toasted soybeans treated with specific enzymes to enhance their digestibility [22,23]. Concurrently, Torrecillas et al. [6] demonstrated that the incorporation of fishmeal (FM) at a rate of 10%, in conjunction with terrestrial meals of animal and plant origin, does not impede growth or compromise the integrity of the intestinal mucosa. As demonstrated in the study by Torrecillas et al. [19], incorporating 5% fishmeal, along with a greater proportion of terrestrial proteins, namely soybean meal, wheat gluten, and blood meal at 20%, 22%, and 7%, respectively, in the diet of seabass has been shown to enhance growth performance, promote intestinal health, and reduce the pro-inflammatory response (*IL-1β*, *COX-2*). Another study, adopting a circular economy approach to partial replacement of fishmeal (FM) with alternative ingredients such as poultry meal (PM) and a mix of poultry oil and DHA-rich microalgae, has shown improvements in production yields, such as fillet quality, in relation to n-3 PUFAs [4,9]. A 30% inclusion of fishmeal (FM) with organic vegetable meals promotes optimal growth performance, digestive function, histological integrity, and fillet quality, without compromising fish health [24]. Conversely, a complete replacement of fishmeal and oil (FM, FO) with by-products from pelagic species, such as sardines (*Sardina pilchardus*) and Atlantic mackerel (*Scomber scombrus*), resulted in a 100% replacement, yielding outcomes that promoted growth, digestibility, and the intestinal microbiota pool of seabass [25]. Using poultry by-products to replace fishmeal had no adverse effects on the growth or intestinal/liver health of European seabass [26]. In addition, the use of mixed protein sources, including insect meal, poultry meal, and vegetable meals, has been associated with satisfactory growth performance. However, feed costs may increase despite the complete replacement of fishmeal.

Research on European seabass has primarily focused on optimizing formulations using single or multiple protein sources, adding synthetic amino acids to meet their requirements and maintain amino acid balance. These strategies have led to the development of different diets aimed at improving productive performance and promoting intestinal health through the incorporation of sustainable ingredients derived from plant, animal, single-celled organism, and livestock by-products. Therefore, the objective of this study was to evaluate the effect of total and partial replacement of fishmeal with plant-based sources on growth performance, gene expression, and gut health, as well as on the economic profitability of diets formulated for European seabass under experimental conditions.

## 2. Materials and Methods

### 2.1. Ethical Aspects

The fish in the tanks were subject to daily inspections. In addition, the fish were weighed individually every four weeks, and their health status was assessed through observation after being sedated with clove oil dissolved in water (10 mg L^−1^ of water) to minimize animal suffering. Animals were euthanized by an excess of clove oil (150 mg L^−1^) and then dissected.

The experimental protocol was conducted in accordance with Royal Decree 53/2013 and the European Directive 2010/63/EU, which stipulates the principles for the protection of animals used for scientific purposes. The protocol underwent a rigorous evaluation and approval process by the Animal Ethics and Welfare Committee of the Polytechnic University of Valencia (cod 2021/VSC/PEA/0268), ensuring adherence to the highest standards for animal welfare and the ethical conduct of research.

### 2.2. Fish and Experimental Conditions

The experiment was conducted at the aquaculture laboratory facilities of the Department of Animal Science at the Polytechnic University of Valencia, Valencia, Spain. Juvenile seabass (*Dicentrarchus labrax*) from the Sonrionansa SL fish farm, located in the autonomous community of Cantabria in Spain, were used for the study.

At the beginning of the experiment, the fish had an average weight of 40 g. A total of 450 seabass were distributed across nine fiberglass tanks, with 50 fish per tank, each with a volume of 1750 L, as part of a saltwater recirculating system with a total capacity of 75 m^3^. The system was equipped with a rotating mechanical filter, a gravity biofilter with a capacity of 6 m^3^, and a skim (March to December).

The fish were fed to visual satiety twice a day, from Monday to Friday (at 9:00 AM and 2:00 PM), and once (at 10:00 AM) on Saturdays, for 196 days. The physical and chemical parameters of the tanks were monitored as follows: dissolved oxygen, 7.62 ± 0.41 mg L^−1^; temperature, 21.53 ± 2.2 °C; ammonium, 0.18 ± 0.24 mg L^−1^; and nitrates, 92. 32 ± 23.38 mg L^−1^, nitrites 0.25 ± 0.11 mg L^−1^, salinity 16.15 ± 2.20 g L^−1^ (salinity 0/00), pH 7.02 ± 0.32.

### 2.3. Experimental Feed

The experimental diets were prepared at the Polytechnic University of Valencia using a Clextral BC45 semi-industrial extruder through the cooking-extrusion process (CLEXTRAL BC-45, St. Etienne, France), whose equipment belongs to the Department of Animal Science. Three isoproteic (46%) and isolipidic (17%) experimental diets were formulated: with different levels of fishmeal inclusion (0, 15 and 30%, named diets 0 FM, 15 FM, and 30 FM, respectively) replaced with a vegetable mixture (wheat, wheat gluten, soybean meal) and including synthetic Tau and Met to meet the nutritional requirements established for European seabass. In addition, lysine and arginine were supplemented in the 15 FM and 0 FM diets to maintain the amino acid balance.

The formulation is centered on optimizing an economically viable and sustainable feed by using the 30 FM treatment as a control diet and evaluating the partial or total replacement of fishmeal with plant-based sources.

The ingredients used in the feed and the composition of the diet are shown in Table 1 and Table 2.

### 2.4. Proximate Composition and Amino Acid Analysis

The nutritional ingredients of the feed (Table 1) and the initial fish were analyzed according to the AOAC [28]: dry matter (at 105 °C until constant weight), ash (incineration process at 550 °C for 5 h), crude protein (determined by the DUMAS direct combustion method—LECO CN628 equipment, Geleen, Netherlands) and crude lipids were extracted with diethyl ether (ANKOM XT10, Macedon, NY, USA) Technology Method 2 (2000). It is imperative to note that each analysis was performed in triplicate.

The amino acid content of the feed (Table 2) and the fish bodies were analyzed using the Waters high-performance liquid chromatography (HPLC) method, which includes two pumps (Model 515; Waters, Milford, MA, USA), a Model 717 autosampler (Waters), a fluorescence detector (Model 474; Waters), and a temperature control, as described by Bosch et al. [29]. After hydrolysis, an internal standard of aminobutyric acid was incorporated. Finally, AQC (6-aminoquinolyl-N-hydroxysuccinimidyl carbamate) was utilized for the derivatization process and to facilitate the quantification of amino acids. Derivatized amino acids form stable derivatives, such as primary and secondary amino acids (those with a free amino group and those whose nitrogen-containing component is bound to the pyrrolidine ring, such as proline). These can be detected by fluorimetry after being separated by HPLC. Alpha-aminobutyric acid was used as an internal standard.

Following the oxidation process with perfluoroacetic acid, cysteine and methionine were identified as cystic acid and methionine sulfone, respectively. The isolation of amino acids was achieved by employing an affinity chromatography process, utilizing the Waters AcQ system with a C18 reverse-phase column measuring 150 mm × 3.9 mm. All analyses were performed twice.

### 2.5. Growth and Nutrient Efficiency Indices

At the conclusion of the study, all treatments were evaluated, with three tanks per treatment; each tank was served as an experimental unit, allowing for the calculation of growth rates and nutritional efficiency indices, including final weight (FW), specific growth rate (SGR), survival (%), feed intake (FI) (feeding days), and feed conversion ratio (FCR): These parameters were calculated using the following equations:

SGR = 100 × ln (final weight/initial weight)/days

FI (g 100 g fish^−1^ day^−1^) = 100 × feed consumption (g)/average biomass (g) × days

FCR = feed consumption (g)/biomass gain (g)

### 2.6. Biometric Indices

To evaluate the biometric indices, five fish per tank were selected and euthanized; this resulted in a total of 15 fish per treatment at the conclusion of the study. An overdose of anesthetic euthanized the subjects, the formulation of which was based on clove oil (150 mg L^−1^) and water. Subsequently, a series of measurements was taken to ascertain the total length (cm), total weight (g), carcass weight (g), viscera weight (g), liver weight (g), and visceral fat weight (g) of the subjects. These measurements were then utilized to calculate various biometric indices, including Condition Factor (CF), Viscerosomatic Index (VSI), Hepatosomatic Index (HSI), and Mesenteric Fat (MF).

CF = 100 × (total fish weight (g)/total length^3^ (cm))

VSI (%) = 100 × (visceral weight (g)/fish weight (g))

HIS (%) = 100 × (liver weight (g)/fish weight (g))

MF (%) = 100 × (mesenteric fat weight (g)/fish weight (g))

Finally, the samples were assigned labels according to the type of treatment they received and stored at a temperature of −30 °C for subsequent analysis.

### 2.7. Retention Efficiency

The efficiency of protein, fat and energy retention was calculated using the following equations, giving rise to the productive protein value (PPV %), productive fat value (PFV %) and productive energy value (PEV %).

$$\mathrm{PPV}\left(\%\right)=\frac{\left[\mathrm{Retained}\;\mathrm{protein}\left(\mathrm{Final}\;\mathrm{fish}\;\mathrm{protein}\times \mathrm{Final}\;\mathrm{biomass}\left(g\right)\right)-\left(\mathrm{Initial}\;\mathrm{fish}\;\mathrm{protein}\times \mathrm{Initial}\;\mathrm{biomass}\left(g\right)\right)\right]}{\left[\mathrm{Protein}\;\mathrm{ingested}\left(\mathrm{kg}\;\mathrm{of}\;\mathrm{food}\;\mathrm{ingested}\times \mathrm{\%crude}\;\mathrm{protein}\;\mathrm{of}\;\mathrm{feed}\right)\right]}\times 100$$

$$\mathrm{PFV}\left(\%\right)=\frac{\left[\mathrm{Retained}\;\mathrm{fat}\;(\mathrm{Final}\;\mathrm{fish}\;\mathrm{fat}\times \mathrm{Final}\;\mathrm{biomass}\left(g\right))-\left(\mathrm{Initial}\;\mathrm{fish}\;\mathrm{fat}\times \mathrm{Initial}\;\mathrm{biomass}\;(g)\right)\right]}{\left[\mathrm{Fat}\;\mathrm{ingested}(\mathrm{kg}\;\mathrm{food}\;\mathrm{ingested}\times \mathrm{\%crude}\;\mathrm{fat}\;\mathrm{of}\;\mathrm{feed})\right]}\times 100$$

$$\mathrm{PEV}\left(\%\right)=\frac{\left[\mathrm{Retained}\;\mathrm{Energy}(\mathrm{Final}\;\mathrm{fish}\;\mathrm{Energy}\times \mathrm{Final}\;\mathrm{biomass}\left(g\right))-\left(\mathrm{Initial}\;\mathrm{fish}\;\mathrm{energy}\times \mathrm{Initial}\;\mathrm{biomass}(g)\right)\right]}{\left[\mathrm{Energy}\;\mathrm{ingested}(\mathrm{kg}\;\mathrm{feed}\;\mathrm{ingested}\times \mathrm{\%feed}\;\mathrm{energy})\right]}\times 100$$

### 2.8. Amino Acid Retention Efficiency

The efficiency of amino acid retention (AARE) and the production values were calculated using the ratio of the nutrients retained to the nutrients ingested. This parameter was calculated using the following equation:

$$\mathrm{AARE}\left(\%\right)=\frac{\left[\mathrm{Final}\;\mathrm{body}\;\mathrm{amino}\;\mathrm{acids}\times \mathrm{Final}\;\mathrm{biomass}(g))-(\mathrm{Initial}\;\mathrm{body}\;\mathrm{amino}\;\mathrm{acids}\times \mathrm{Initial}\;\mathrm{biomass}(g)\right]}{\left[\mathrm{Feed}\;\mathrm{Intake}(g)\times \mathrm{Dietary}\;\mathrm{Amino}\;\mathrm{Acids}(\%)\right]}\times 100$$

### 2.9. Gene Expression

In the case of gene expression in the intestinal tract of seabass, a total of 27 fish were sampled (3 fish per tank, nine fish per treatment). The samples collected were a section of the posterior intestine (PI) from each fish, which was then placed in an RNAlater solution (Qiagen, Barcelona, Spain) and stored at 4 °C for 24 h. The samples were then stored at −20 °C until the gene expression analysis process.

#### 2.9.1. tRNA to cDNA Extraction

For the extraction of total ribonucleic acid (tRNA) from tissues of the posterior portion of the intestine, a phenol/chloroform-based approach was adopted, complemented by TRIzol reagent (Invitrogen, Barcelona, Spain), as per the manufacturer’s guidelines. This was followed by the implementation of recombinant DNase I, RNase-free (Roche, Penzberg, Germany), in accordance with the manufacturer’s protocol. The NanoDrop 2000c spectrophotometer (Thermo Fisher Scientific, Madrid, Spain) was used to determine the quality of the extraction (purity) and concentration of the total RNA. During the analysis, only results with an A260/280 ratio ranging from 1.8 to 2 and an A260/230 ratio greater than two were considered. To prevent tRNA degradation, all samples needed to be stored at −80 °C to maintain their integrity and facilitate subsequent analysis during the complementary deoxyribonucleic acid (cDNA) synthesis process.

Subsequently, in accordance with the manufacturer’s guidelines and utilizing the Applied Biosystems 2720 thermal cycler, cDNA was synthesized employing the qScript Flex cDNA kit (Quanta BioScience, Beverly, MA, USA) and 1 μg of tRNA. The thermal cycler conditions were as follows: The temperature was increased to 22 °C for 5 min, followed by a 42 °C temperature for 30 min, and then to 85 °C for an additional 5 min. The cDNA was stored at −20 °C until further analysis of gene expression was performed.

#### 2.9.2. Selection of Reference Genes and qPCR

All assays were performed using LightCycler^®^ 480 SYBR^®^ Green I Master Reagent (Roche, Penzberg, Germany) and the LightCycler^®^ 96 Instrument (Roche, Madrid, Spain). The final volume added to each well was 10 µL, comprising 3 µL of cDNA (prepared at a dilution of 1:20), 1 µL of each forward and reverse primer (at a concentration of 5 µM), and 5 µL of 2× LightCycler 480 SYBR Green I Master (Roche, Barcelona, Spain). Gene expression was examined in 96-well plates, and each PCR reaction was performed in duplicate. Finally, a group of samples was used as a calibrator for the three diets with a 1:20 dilution.

For each gene, a calibrator sample was prepared by pooling equal aliquots of cDNA from all experimental samples, thus representing all treatments and experimental fish. The pooled cDNA was diluted 1:64 and included in each qPCR plate as an inter-plate calibrator. The same calibrator was used for all plates corresponding to a given gene to assess and correct for inter-plate variability and to ensure comparability of gene expression measurements across plates.

According to research conducted in the context of RT-qPCR analysis, three reference genes have been identified: glyceraldehyde-3-phosphate dehydrogenase (*GADPH*), elongation factor 1α (*EF1α*), and *β-actin* (*β-act*) [30,31]. The BestKeeper program Version 1 was utilized for this procedure, and values with a standard deviation of less than 1 were considered reference genes [32]. After stability analysis, which was performed using the BestKeeper Programme, statistically significant differences (*p* < 0.05) were found between treatments. For both genes, *β-act* and *EF1α*, the standard deviations were 1.66 and 1, respectively, and the geometric means were 22.35 ± 3.16 and 21.21 ± 2.0, respectively, with *EF1α* being selected as the reference gene. The reference and differential genes were presented in Supplementary Figure S1.

Furthermore, 16 differential genes (Table 3) were identified by the RT-qPCR method due to their importance in inflammation and immune response processes. The regulatory mechanisms that control the immune response and inflammatory processes are known as cytokines [33,34,35]. Examples of such cytokines include interleukin-1β (*IL-1β*), interleukin-8 (*IL-8*), interleukin-6 (*IL-6*), and interleukin-10 (*IL-10*). It has been established that certain genes function as inflammatory markers, including transforming growth factor beta (*TGF-β*), tumor necrosis factor-alpha (*TNF-α*), and cyclooxygenase-2 (*COX-2*) [35]. It is essential to recognize the crucial role these genes play in the process of apoptosis under stress conditions, as demonstrated by the research of Reis et al. [36] and Takle & Andersen [37]. It is evident that other genes are implicated in adaptive immunity, and these have been attributed to T cell antibodies (*TCR β*), T cell co-receptors (*CD4* and *CD8α*), and immunoglobulin (*Ig*), to protect the intestinal epithelium, as evidenced by *Muc2* [16]. Furthermore, those related to the storage and transport of iron, which is associated with the immune response, are ferritin (*Fth*) and transferrin (*Trf*) [38]. Table 3 presents the 16 reference genes analyzed.

The variability of gene expression and the most predominant genes are indicative of the biological aspect of the fish. Subsequent analysis and selection of genes exhibiting Ct values ranging from 26 to 35, with *Casp9*, *TCR β*, *IL-8*, *IL-10*, *TGF β*, *TNF α*, *Muc2*, and *Fth* being selected for further investigation. Conversely, the expression values of *CD4*, *IL-6*, *Casp3*, and *Ig* exhibited marginal similarity. However, the *Trf*, *IL1β*, *COX2*, and *CD8α* genes demonstrated elevated Ct values, suggesting minimal expression levels. Consequently, this group of eight genes was excluded from subsequent analyses (Supplementary Figure S2, Real-time quantitative polymerase chain reaction (RT-qPCR) for each differential gene using sample pools in European seabass fed experimental diets).

#### 2.9.3. Relative Quantification

The *EF1α* reference genes and the use of calibrators on different plates were established for relative quantification of differential gene expression, which minimized differences and performance between plates. The subsequent analysis of the results was conducted in accordance with the 2^−∆∆Ct^ method [43], the formula for which is as follows:

Normalized ratio (N_R_) = *E*_T_ ^CtT(C)–CtT(S)^ × *E*_R_ ^CtR(S)–CtR(C)^

where

*E*_T_: The efficiency of the differential gene is the focus of this study.
C: Calibrator.
S: Sample of the gene of interest.
*E*_R_: Efficiency of the reference gene.
CtT: Number of cycles in which the threshold is detected in the differential gene.
CtR: Number of cycles in which the threshold is detected in the reference gene.

Efficiencies were calculated based on standard curves, with specific serial dilutions [44]. However, the calculation was performed for both the study genes and the reference genes.

As illustrated in Supplementary Table S1, the efficiencies range from 1.85 to 2.1. Concurrently, disparate values of amplicon, slope, R^2^, and efficiency are evident for the genes tested and their standard curve.

The genes used with the different calibrators described in Supplementary Figure S3 are shown. The relative quantification of a single calibrator was expressed using the mean, represented by a single bar for the differential genes (*IL-10*, *TGF-β*, *IL-8*, *Muc2*, *Casp9*, *β-actin*) as well as the reference gene (*EF1α*). For the *TCR β*, *TNF α*, and *Fth* genes, their expression required different plates to be measured using the mean of two calibrators. Meanwhile, slight variations were observed. Furthermore, as shown in the Supplementary Materials in Figure S3, variation between plates is minimal for these latter genes; therefore, human error did not play a significant role in that variation.

### 2.10. Economic Analysis

The cost-benefit analysis for this study was determined by calculating the economic conversion rate (ECR) and the economic profit index (EPI), which were adapted by Martínez-Llorens et al. [45]. The total price of the diet per kilogram was calculated based on the reference cost of each input per metric ton (MT) for 2025.

E.C.R = (EUR/kg diet) = FCR × Diet Price

EPI (€ fish^−1^) = [weight gain (kg) × selling price (€ kg^−1^)] − [weight gain (kg) × diet price (€ kg diet^−1^)]

### 2.11. Statistical Analysis

Growth parameters, biometric data, and the retention efficiency (of nutrients and amino acids) were evaluated with analysis of variance (ANOVA), with a Newman-Keuls test. Initial weight was used as a covariate. Means were compared, and significant differences between diets (at a level of *p* < 0.05 and a confidence interval of 95%) were observed. The Statgraphics Plus 5.1 program (Statistical Graphics Corp., Rockville, MD, USA) was utilized, and the results were expressed as the mean ± standard error of the mean. The use of statistical software facilitated the verification of normality and homogeneity of variances for all data sets.

For the analysis of gene expression data, SAS 9.4 software (SAS Institute Inc., Cary, NC, USA) was utilized, and a one-way analysis of variance was performed, with the diet effect considered as the primary component. The GLM procedure was employed for variables with a normal distribution. In contrast, the GLIMMIX procedure was used for variables with a non-normal distribution (relative expression of *Muc2* and *Fth*), which had a Poisson distribution. Statistical significance was attributed to differences when *p* < 0.05; results are expressed as the mean ± standard error.

## 3. Results

### 3.1. Growth and Nutritional Parameters

During the 196-day experimental period, a significant increase in weight was observed in European seabass fed the different diets (Figure 1). However, lower fish weights were observed for the 0 FM treatment compared to the 15 FM and 30 FM treatments from the third month of evaluation onwards.

In Table 4 significant differences between the three treatments are shown. The 30 FM diet resulted in a higher final weight (347 g) and a higher specific growth rate (1.09% day^−1^), while the 0 FM diet had a lower final weight and specific growth rate (269.49 g; 0.99% day^−1^, respectively). Survival rates were higher in the 30 FM and 15 FM groups (93.33% and 92.67%, respectively) than in the 0 FM group (84%) and tended to be lower. No significant differences were found for feed intake and food conversion ratio.

### 3.2. Biometric Parameters of Seabass Fed Experimental Diets

As illustrated in Table 5, the biometric indices measured at the end of the evaluation period showed statistically significant differences for condition factor (CF), hepatosomatic index (HSI), viscerosomatic index (VSI), and mesenteric fat (MF) (*p* < 0.05). The lowest values for CF, VSI, and MF corresponded to the 30 FM diet (1.34 g cm^−3^, 11.82%, and 5.26%, respectively). However, this trend was not observed for HSI, which showed the highest value in the 30 FM group compared to the other treatments.

### 3.3. Body Composition and Nutrient Retention Efficiency

The results of the body composition of both the initial and final fish, as well as the retention efficiency of the seabass fed the experimental diets, are presented in Table 6. Statistically significant differences were observed only in dry matter (DM) and fat productive value (FPV) efficiency (*p* < 0.05); the highest values were observed in the 15 FM treatment (39.56% and 84.25% respectively).

### 3.4. Amino Acid Retention Efficiency

The results of amino acid retention are shown in Table 7. The study revealed statistically significant differences (*p* < 0.05) only for arginine and threonine among the essential amino acids in fish fed different levels of fishmeal. The highest arginine retention values were observed in the 30 FM treatment group, whereas the 0 FM group exhibited the lowest values (32.40% and 19.44%, respectively). In contrast, threonine retention was highest in the 0 FM diet (46.96%). Meanwhile, in the case of non-essential amino acids, statistically significant differences (*p* < 0.05) were observed for alanine, aspartic acid, cysteine, and glycine. The highest retention values for alanine, aspartic acid, and glycine were identified in fish fed the 0 FM diet (70.66%, 52.43%, and 72.39%, respectively). Conversely, the highest cysteine retention value (20.37%) was observed in fish fed the 30 FM diet.

The final amino acid retention values (Table 7) are more relevant for studies on growth performance in sustainable feed. For fillet quality, complementary analyses of palatability and nutritional value are needed, which are beyond the scope of this research. This does not detract from the merit or importance of the present study.

### 3.5. Intestinal Health and Gene Expression

The relative expression of genes associated with intestinal health and immune response in European seabass fed the experimental diets is presented in Table 8 and Figure 2. No statistically significant differences (*p* > 0.05) were observed for the expression of *Casp9*, *TCR β*, *IL-8*, *IL-10*, *TGF-β*, *TNF-α*, and *Fth* among dietary treatments. However, significant differences (*p* < 0.05) were detected for the expression of *Muc2* (Figure 2).

Fish fed the 15 FM diet exhibited the highest expression levels of *Muc2* (22.527), whereas no significant differences were detected between fish fed the 30 FM and 0 FM diets. Although *TNF-α* expression showed higher numerical values in fish fed the 30 FM diet (0.078), and *TGF-β* expression was numerically higher in fish fed the 15 FM diet (0.013), these differences were not statistically significant (*p* > 0.05). Likewise, *IL-8* expression tended to increase with increasing levels of fishmeal replacement, reaching the highest value in fish fed the 0 FM diet (0.215), although no statistically significant differences were detected among treatments.

### 3.6. Economic Analysis

A cost-benefit analysis was conducted for the study, and the results are presented in Table 9. A statistically significant difference (*p* < 0.05) was observed in the economic conversion ratio (ECR), which was higher for the 0 FM treatment (EUR 2.07 kg^−1^). In comparison, the economic profit index (EPI) was higher for the 30 FM treatment (EUR 1.34 fish^−1^). These disparities in the ECR can be attributed to the elevated cost of the feed. As the proportion of fishmeal in the diet diminishes, the proportion of vegetable protein increases. Conversely, a lower EPI value would indicate a lower number of fish at the end of the growth trial relative to treatment 0 FM.

## 4. Discussion

### 4.1. Fish Performance

In search of sustainable aquaculture for enhanced profitability, it is imperative to comprehend and optimize the design of sustainable feed, evaluating the benefits of various protein ingredients while ensuring high-quality technological design. This process involves incorporating these ingredients into the formulation to partially or fully replace fishmeal without compromising fish growth and health.

Consequently, feed constitutes a substantial proportion of the total cost of aquaculture production, accounting for 40–60% of the total. Consequently, as a preliminary measure to curtail the use of fishmeal and fish oil in aquaculture, diverse plant protein sources were employed, culminating in a substantial reduction in fishmeal [10,12,22]. However, it is imperative to consider the pretreatment of these sources to reduce antinutritional factors that hinder nutrient absorption, inhibit digestive enzymes, and affect the health of the fish’s liver and intestine [16,17,18,20,31]. Several studies on European seabass have sought to mitigate the problems that can arise from increasing the proportion of plant-based protein sources in the diet without prior pretreatment. These studies have focused on so-called fermented feeds, which increase the nutritional value of plant-based feeds, promoting good growth outcomes without compromising growth and achieving reductions of 18–20% in fishmeal [46,47,48,49,50]. Nevertheless, certain studies have yielded unfavorable outcomes, prompting recommendations to enhance palatability to stimulate increased voluntary intake [51].

In the present study, the incorporation of plant-based alternatives into fishmeal formulations was investigated to reduce the fishmeal content to a maximum of 15% (15 FM). The results demonstrated that this substitution did not adversely affect growth performance, specific growth rate (SGR), or feed conversion ratio (FCR) compared with the 30 FM diet. As demonstrated in other studies, an increase in the levels of plant-based sources and the incorporation of hydrolyzed animal protein into the diet have been shown to result in a reduction of fishmeal to levels below 10% in balanced diets for European seabass [52,53,54]. Consequently, when increasing the levels of plant protein, sources of essential amino acids such as methionine, lysine and arginine must be included, as they are absent in these sources, without affecting the performance and health of the fish [9,12,55].

It has been demonstrated that increasing the proportion of plant protein in feed, to produce animals that exceed commercial size, results in a reduction of digestive capacity, impaired growth performance and feed efficiency, and a negative impact on intestinal integrity and product quality [51,54,56,57,58]. This dietary modification, in the absence of fishmeal and with an augmented proportion of vegetable sources, has been shown to enhance the levels of insoluble fiber, thereby accelerating intestinal transit and decreasing nutrient absorption [55,59,60,61].

Wassef et al. [62] reported the results of a study on juvenile seabass fed a diet containing 20% fishmeal, along with other plant-based ingredients (corn gluten, soybean meal and concentrate, corn meal, and wheat flour), and taurine. The study found that the fish achieved good final weights (FW) and specific growth rates (SGR). Concurrently, the feed conversion ratio (FCR) was consistent with the present study’s findings for the 30 FM treatment (30% fishmeal). In contrast, Rodrigues-Dos-Santos et al. [52] achieved higher feed conversion ratios (FCR) of 1.1 and a condition factor of 1.22 in juvenile seabass, which exceeded the results of the present study. This discrepancy can be attributed to the diet, which comprised plant-based sources. It has been demonstrated that incorporating terrestrial sources is instrumental in achieving optimal growth rates, thereby contributing to the observed disparities in growth performance. However, studies employing plant-based sources with varying degrees of fishmeal (FM) replacement in seabass have revealed substantial variations in weight gain, specific growth rate (SGR), and feed conversion ratio (FCR) [8,9,25,49,62,63,64].

Animal-derived proteins, such as poultry byproducts (PBM), have been identified as a potentially viable source of nutrition for European seabass. These proteins have been shown to positively impact the health and growth of fish while also contributing to intestinal homeostasis [4,5,26,65]. Specifically, the partial or complete replacement of fishmeal with these animal-derived proteins is beneficial in this context. Conversely, insect meal (IM) has been shown to substitute for up to 30% of fishmeal. However, its incorporation into the diet can impact lipid metabolism and the fatty acid profile of the fillet, particularly at higher levels [4,11,65,66,67,68,69]. The specific effects of IM on the fillet depend on the type of substrate used to feed the insect larvae. The use of protein sources from diverse origins, including animal byproducts, plant-based materials, and insect-based sources, is poised to reduce reliance on fishmeal further, thereby exerting a more substantial influence on the circular economy. Nevertheless, the sustainability and cost-effectiveness of insect meal remain constrained by current limitations [4,5,26,65].

A more cost-effective alternative would be to work with one or two sustainable protein sources, avoiding economic fluctuations and shifting towards a circular economy approach. This study demonstrated a 15% reduction in fishmeal, resulting in promising specific growth rates and feed conversion ratios (SGR, FCR) compared with other studies on seabass using plant-based sources, which reduced fishmeal by 20–30%.

### 4.2. Final Biometric Parameters

In contrast, biometric indices (the viscerosomatic index, the hepatosomatic index, and mesenteric fat) exhibited elevated values in the presence of plant-based sources, contingent on the level of fishmeal (FM) incorporated into the diet and the timing of the assessment. This finding provides a rationale for the observed partial increase in the VSI and mesenteric fat in seabass exposed to a reduced level of fishmeal. Conversely, the hepatosomatic index declined gradually as the proportion of fishmeal was reduced (2.16; 1.91; 1.83%; 30% FM, 15% FM, and 0% FM, respectively) in the experimental treatments. Research has demonstrated that this results in specific changes in carbohydrate (insoluble fiber) and lipid metabolism, thereby undermining immunosuppression [6,60]. Research has also examined the impact of dietary regimens on fish survival. Research has demonstrated that specific dietary components, such as antinutritional factors (ANFs), and the deficiency of essential amino acids (Lys, Met) in feeds with higher vegetable protein content, can influence fish growth and survival [15,17,22,52,53,62,63].

### 4.3. Body Composition and Retention Nutrient Efficiency

No significant differences (*p* > 0.05) were found between the experimental diets in terms of body composition. However, significant differences were observed in the dry matter composition of the European seabass body in the 15 FM (15% fishmeal) treatment (*p* < 0.05). Furthermore, the type of protein source in the seabass’s diet may slightly influence its proximate composition and protein retention [52,53,63,70]. Some studies have shown that replacing 50% of the fishmeal with animal protein sources has no significant effect on whole-body protein and lipid content [68,71]. However, other studies using a combination of animal by-products and plant protein sources have shown that up to 50% of the fishmeal can be replaced without compromising weight gain. Some differences have been observed in whole-body protein, lipid, and ash content when fishmeal inclusion levels range from 200 to 250 g kg^−1^ in the diets [26,72,73]. Nevertheless, these levels of fishmeal inclusion are higher than in the present study.

In our study, no significant differences (*p* > 0.05) were found in protein- or energy-productive values (PPV and PEV, respectively). However, significant differences in the productive value of fat (PFV) were observed, with treatment 15 FM showing the greatest fat retention. These findings are consistent with those reported by Kaushik et al. [10], who found that greater inclusion of plant-based sources led to greater fat deposition in seabass. In contrast, Marzouk et al. [26] reported a PPV of 33.23%, a higher value, and replaced 50% of fishmeal with terrestrial animal protein by-products.

### 4.4. Retention Amino Acid Efficiency

Statistically significant differences (*p* < 0.05) in the retention efficiency of essential amino acids were only observed for arginine (Arg) and threonine (Thr). The 30 FM diet, containing 30% fishmeal, showed the highest retention rate (31.40%), compared to the 0 FM diet (19.44%), which contained no fishmeal. This was attributable to the necessity of compensating for arginine deficiencies in the diet when it was not supplied, in contrast to the 0.5–1% arginine supplied in the 15 FM and 0 FM diets, respectively. As fishmeal is replaced to a greater extent, arginine (Arg) retention levels decrease; nevertheless, this would affect the growth of fish fed a diet containing 0% fishmeal. Conversely, other studies using a 60% inclusion of a mixture of plant sources found no effect on growth, palatability, and fillet quality [74]. However, the opposite was observed for threonine (Thr): retention efficiency was higher for the 0 FM diet. It was attributed to an increased demand for muscle tissue formation and greater metabolic efficiency, due to the reduction or absence of fishmeal in the diet [10,64]. Consequently, a deficiency of amino acids in seabass diets resulting from the total replacement of fishmeal with plant-based sources led to reduced growth (0 FM). Regardless of the fish species or the type of sustainable ingredient used to partially or totally replace fishmeal, the amino acid composition of structural fish protein remains unchanged [6,19,57,62,64].

Research into feeding seabass diets that are high in plant-based ingredients as an alternative to fishmeal (FM) has revealed imbalances in the diet, emphasizing variations in the retention of essential amino acids [24,46,64,70]. Similar variations in retention levels were observed in diets containing a combination of plant and animal proteins [26,68,71]. Although the formulation of seabass feed is becoming more optimized using more sustainable protein sources (terrestrial and plant-based), processed (hydrolyzed) by-products from aquatic species are currently being incorporated. However, their inclusion level is set at 2.5% to 5% of the diet, thereby reducing the inclusion of fishmeal by up to 15% [52,75,76]. It would help to prevent imbalances in protein metabolism, low body weight, reduced feed intake, and intestinal damage.

### 4.5. Gut Health and Gene Expression

Research into the nutrition of carnivorous fish, particularly seabass, indicates that excessive supplementation with plant-based proteins can stimulate inflammatory and immune responses in the body by reducing the primary source of animal protein, such as fishmeal [22,57,64,77]. These reactions are caused by antinutritional factors which, if not pretreated, can affect intestinal health and lead to poor nutrient absorption and inhibited intestinal tract enzymes [13,16,31,53,55,78,79,80]. These factors regulate the secretion and transport of hormones, as well as the proliferation of caliciform cells. Additionally, alter the permeability of cell membranes and the absorption of foreign substances (allergens) into the body of the fish, or act as immunostimulants in cytokine production during the early stages of inflammatory processes [16,26,64,70,79].

In the present study, specific upward trends in gene expression were observed (Table 8), although no significant differences between the treatments were found (*p* > 0.05). Consequently, increased expression of the *IL-8* gene was observed in the 0 FM treatment group (0% fishmeal) compared to the 30 FM and 15 FM groups (30% and 15% fishmeal, respectively). This gene and others are involved in the inflammatory response observed during the acute phase, particularly in diets with higher levels of plant protein. In such diets, it acts as a chemotactic agent, mobilizing white blood cells (neutrophils, basophils, T cells, and eosinophils) towards sites of inflammation in the intestinal mucosa [6,7,19,81,82]. Notable observations were made in different regions (anterior and posterior) of the intestine of another Mediterranean species, the gilthead seabream (*Sparus aurata*). In this species, certain trends towards greater induction of inflammatory and immunological markers (*IL-1β*, *COX2*, and *IgM*) were observed in the anterior region of the intestine following a mixed diet containing a plant-based protein source and a 15% marine ingredient meal [16]. In contrast, when European seabass were fed diets containing 20% fishmeal and 30% soybean meal, there was only a slight decrease in caliciform cells in the posterior region of the intestine [83]. Parma et al. [84] reported that, at an inclusion level of 10% fishmeal (FM) and 5% bioactive peptides from salmon by-products (BP5), there was a reduction in the pro-inflammatory markers *IL-1β* and *IL-8*. In contrast, a 5% reduction in fishmeal and a 10% increase in bioactive peptides (BP10) increased the expression of pro-inflammatory markers (*IL-1β* and *IL-8*). Meanwhile, Torrecillas et al. [8], based on genotype-nutrition interactions in wild and selected seabass, reported an increase in inflammatory cytokines (particularly *IL-1β*) and larger goblet cells in the distal intestinal region in wild seabass fed a diet containing 20% fishmeal.

In the present study, higher *TGF-β* expression was observed in the group fed a diet containing 15% fishmeal than in those fed diets containing 30% or 0% fishmeal. It suggests that the 15% fishmeal diet promotes a return to normal by reducing inflammation. In contrast, the 0 FM group showed signs of immunosuppression and an attenuated anti-inflammatory response, leading to reduced *TGF-β* expression. This multifunctional, inhibitory cytokine is associated with the differential gene group [70,85].

The 15 FM treatment showed higher relative expression of the *Muc2* and *β-actin* genes compared to the 30 FM and 0 FM treatments, indicating increased secretion of intestinal mucin by caliciform cells in the posterior region of the seabass intestine. However, a high substitution of fishmeal has been shown to trigger an inflammatory response [26]. Conversely, short-term feeding with plant-based protein sources would be a viable option without adversely affecting feed intake, histology, or intestinal integrity [86]. In juvenile seabass fed diets containing 10% fishmeal and a higher proportion of plant-based protein, increased expression was observed for four of the seven target genes (*IL-1β*, *IL-8*, *IRF1*, and *TNF-α*) involved in mucosal protection and the intestinal inflammatory response. No negative effects on growth were observed due to the inclusion of a feed additive (sodium butyrate) to enhance the sustainability of the feed formulation [41]. While studies have examined inflammatory processes in the intestines of carnivorous species when fishmeal is replaced with plant protein sources, and increased *Muc2* gene expression in *Sparus aurata* has been observed [16], this suggests that seabass fed a 0 FM diet experienced immune compromise due to chronic stress and an extended feeding period, resulting in a 16% increase in mortality by the end of the study. Consequently, from the perspectives of ingredient sustainability, gut health, and fish welfare, it is essential to understand this innate immune organ, which modulates feed utilization and, depending on the nature of the ingredient, affects nutrient absorption.

The *β-actin* gene showed the same pattern as *Muc2*, with higher expression in the 15 FM treatment and lower expression in the 0 FM treatment, suggesting that prolonged stress can affect its expression. While studies in fish with various diets suggest the *β-actin* gene remains constant, it plays a key role in normalizing results [87,88]. *Muc2* is used as a biomarker or a target gene, reflecting its role in immune health and protection against dietary changes and pathogens [87,89,90,91]. Therefore, its expression might be affected by an inflammatory process, as intestinal cells are affected by a higher plant-based diet and lower fishmeal. From a sustainable feed perspective, the 15 FM treatment might be better for intestinal health and performance efficiency, while the 0 FM treatment could reduce survival.

The research results show that a sustainable diet based on plant proteins, containing 15% less fishmeal, improves the productivity, survival, and growth time of European seabass. A reduced inflammatory response and decreased expression of intestinal mucin genes (*Muc2*, *β-actin*, and *IL-8*) were also observed. Recent studies have focused on reducing fishmeal content to as low as 20% by using a mixture of plant- and animal-based protein sources, as well as by-products from marine species. This research has demonstrated positive outcomes in growth performance and intestinal health.

### 4.6. Economic Analysis

Ennayer et al. [73] demonstrated that substituting fishmeal with plant-derived animal protein improved weight gain and the economic benefit index (EPI €/kg) when replacing 50% of fishmeal with marine animal protein, reducing the seabass diet’s fishmeal content by up to 15%.

The study evaluated only plant-based sources and found favorable results with 30% and 15% inclusion levels of fishmeal for weight gain and feed conversion ratio, respectively. Slight differences in feed cost were observed; a greater reduction in fishmeal led to higher feed costs due to higher levels of wheat gluten and synthetic amino acids, such as lysine and arginine. On the other hand, Martínez-Llorens et al. [92] reported very low diet costs, but with a higher fishmeal content (370–195 g kg^−1^). While Sánchez-Lozano et al. [93] reported good long-term weight gain and a lower diet cost (0.90 € kg^−1^) with a moderate level of fishmeal inclusion (533 g kg^−1^), these studies using plant-based sources cannot be compared to the current diet cost due to the significant time gap and fluctuating raw material prices. Meanwhile, Moutinho et al. [94] reported a lower diet cost (0.81 € kg^−1^) when 50% of fishmeal was replaced with meat-and-bone meal, because no synthetic amino acids were added, unlike in the present study, which did include them, which would explain this variation in costs.

The ECR values (2.07–1.65 € kg^−1^) obtained in this study showed significant variation due to greater substitution of fishmeal with plant-based protein sources. In contrast, in the case of sea bream fed a mixed diet with an optimal inclusion level (289 g kg^−1^) of fishmeal and soybean meal, the ECR value was similar to that of the 0 FM treatment without fishmeal. This suggests that it is possible to optimize and improve the design of a more sustainable feed without compromising weight gain [45]. On the other hand, Martínez-Llorens et al. [92] and Moutinho et al. [94] reported lower optimal ECR values (0.88 and 1.24 € kg^−1^, respectively) than those reported in the present study. The economic profit index (EPI) showed that 30% fishmeal was the best treatment, but no differences were observed in weight gain or feed conversion ratio (FCR) with 15% fishmeal. This suggests that the observed disparities were only attributable to variations in diet cost. Conversely, certain studies on EPI have documented a lower value (€1.15 fish^−1^) and a higher value (€1.43 fish^−1^) in economic profitability, under the assumption that the optimal levels of fishmeal inclusion were higher (195–533 g kg^−1^, respectively) than in the present study [92,93]. Martínez-Llorens et al. [45] highlighted that the optimal levels of fishmeal inclusion were 31% and 28.8% in diets for sea bream using plant-based sources, yielding EPI values of 0.36 and 0.64 € fish^−1^. Conversely, using 15% instead of 30% fishmeal in diets for European seabass would reduce the ecological footprint. However, longer feeding periods and feeding beyond market weight compromise intestinal integrity and increase the expression of proinflammatory genes at 15% fish meal, whereas 30% fish meal mixed with plant-based ingredients is a second option for ration sizes larger than the commercial weight and the one with the highest economic return (€1.34 fish^−1^).

Consequently, this research study provides a scientific basis for future discussion on seabass, to evaluate the benefits of the formulated feed, develop a better strategy to balance weight gain and economic profitability in aquaculture production, and determine the types of ingredients to be used in the formulation.

## 5. Conclusions

In the context of sustainability and optimization of feed for European seabass utilizing plant sources, it is advised to incorporate fishmeal (FM) at a proportion of 30–15% of the diet. This approach has demonstrated potential without compromising production performance or survival, up to the point of achieving a satisfactory commercial size. Additionally, it is imperative to supplement essential amino acids in diets comprising a high proportion of plant sources and a low proportion of fishmeal, in order to meet the requirements of the species under study and to avoid imbalances in the retention efficiency of those amino acids that are linked to muscle tissue formation and fat oxidation. Conversely, the long-term replacement of fishmeal with plant protein sources at high or total levels has been shown to induce an inflammatory response, resulting in alterations in intestinal health and the integrity of caliciform cells within the intestinal lumen due to depletion of protective mucus. In turn, it has been shown to increase vulnerability to nutrient absorption problems, compromising the growth and survival of fish.

## Figures and Tables

**Figure 1:**
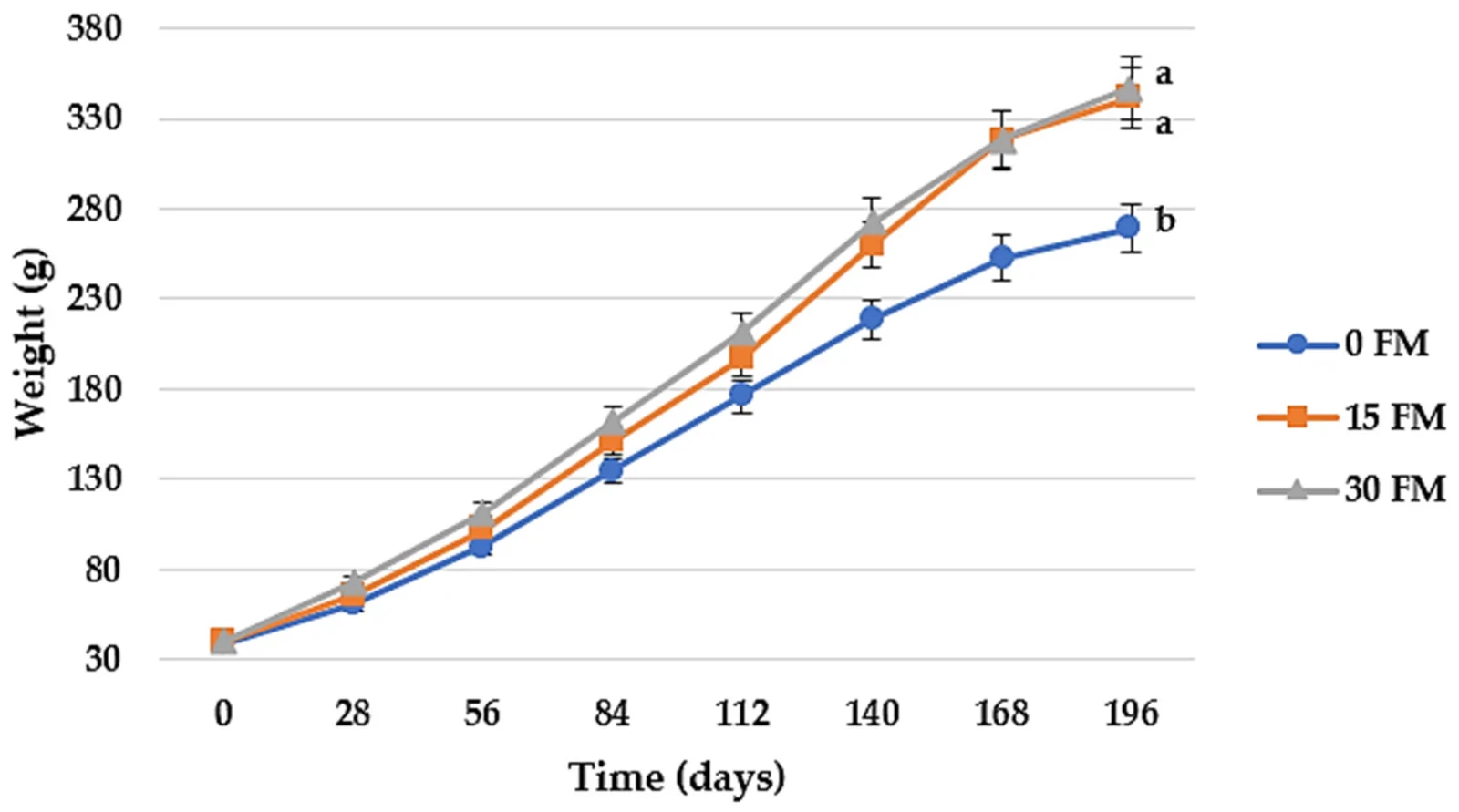
Means of live weight of European seabass development during the 196-day experiment. Statistically significant differences (*p* < 0.05) are indicated by different letters among treatments at each sampling point. Values are presented as means (n = 3).

**Figure 2:**
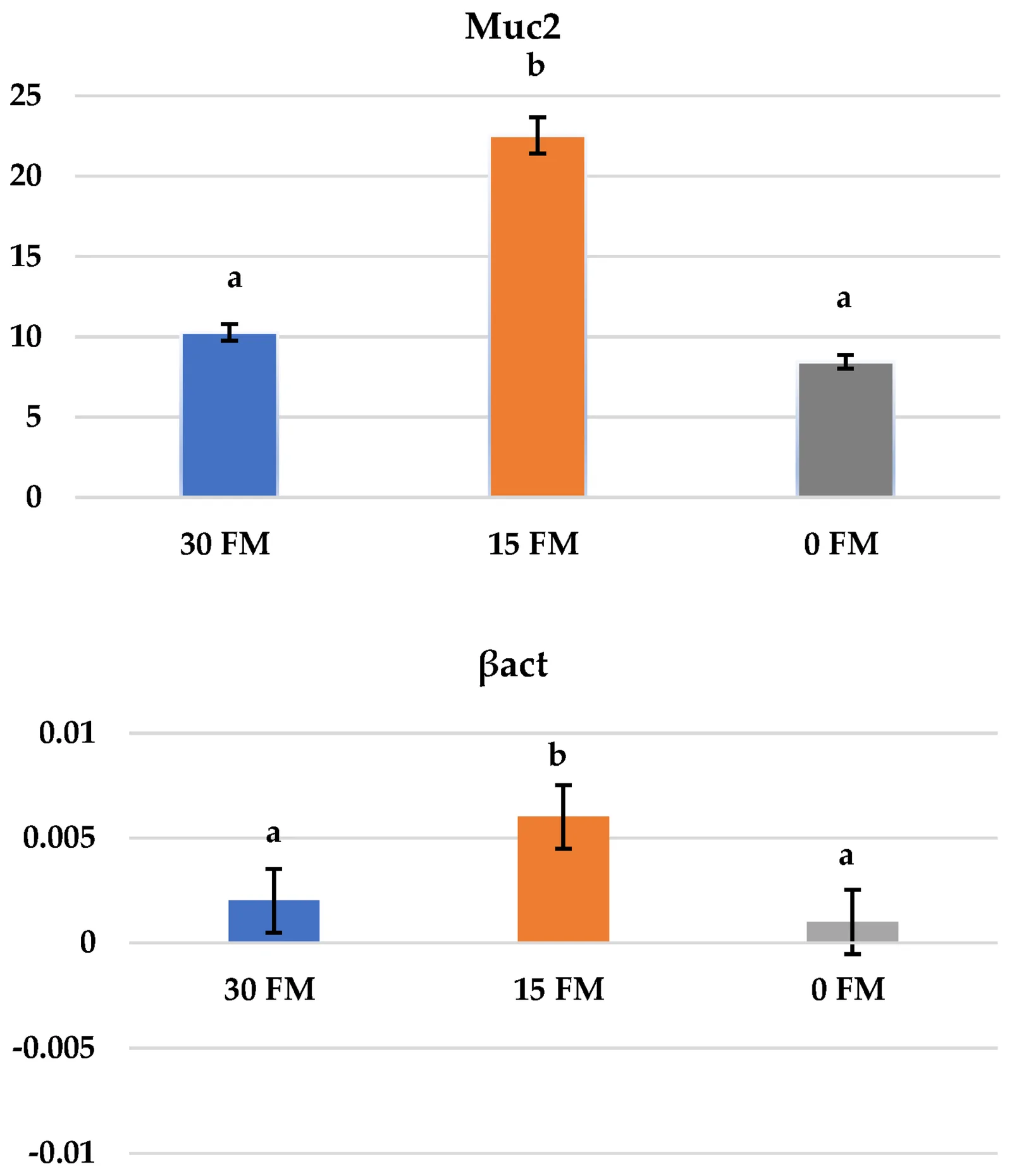
Relative gene expression, different letters indicate significant differences (*p* < 0.05).

**Table 1:** Ingredients and nutritional composition of the experimental diet.

	30 FM	15 FM	0 FM
Ingredients (g kg^−1^)			
Fishmeal	300	150	-
Wheat	179	154	127
Wheat Gluten	122	213	303
Soybean Meal	218	248	277
Soybean Oil	72	72	72
Fish Oil	71	84	97
Calcium Phosphate	20	41	62
Taurine	5	10	20
Methionine	3	6	8
Lysine	-	7	14
Arginine	-	5	10
Vitamins and Minerals ^1^	10	10	10
Nutritional Composition (% Dry matter)			
Dry Matter	91.83	92.26	92.98
Crude Protein	47.24	48.17	46.77
Crude Fat	17.45	17.17	17.67
Ash	9.09	8.36	7.87
Gross Energy (KJ g^−1^) ^2^	24.3	24.2	23.9

^1^ Mix of vitamin and mineral supplementation (g kg^−1^): Premix: 25; Choline, 10; DL-a-tocopherol, 5; ascorbic acid, 5; (PO_4_)_2_Ca_3_, 5. Premix composition: retinol acetate, 1,000,000 IU kg^−1^; calciferol, 500 IU kg^−1^; DL-a-tocopherol, 10; sodium bisulfite menadione, 0.8; thiamine hydrochloride, 2.3; riboflavin, 2.3; pyridoxine hydrochloride, 15; cyanocobalamin, 25; nicotinamide, 15; pantothenic acid, 6; folic acid, 0.65; biotin, 0.07; ascorbic acid, 75; inositol, 15; betaine, 100; polypeptides 12. ^2^ Gross Energy (KJ g^−1^) = [51.8 × (%C/100) − (19.4 × (%N/100)]. Calculated according to Brouwer [27].

**Table 2:** Amino acid composition of the experimental diets.

	30 FM	15 FM	0 FM
EAA ^1^			
Arginine	3.39	3.86	3.58
Histidine	1.00	0.81	0.81
Isoleucine	1.47	1.24	1.08
Leucine	3.24	3.11	2.45
Lysine	3.68	2.78	2.38
Methionine	1.16	1.06	1.05
Phenylalanine	1.80	1.78	1.76
Threonine	1.98	1.50	1.28
Valine	2.01	1.60	1.32
NEAA ^2^			
Alanine	2.74	2.14	1.66
Aspartic acid	4.29	3.68	3.09
Cysteine	0.61	0.62	0.67
Glutamic acid	7.64	9.66	10.86
Glycine	2.44	2.28	2.09
Proline	2.3	2.72	3.49
Serine	2.11	1.96	1.87
Tyrosine	1.50	1.02	1.02

^1^ EAA, essential amino acids (g 100 g^−1^ dry weight); ^2^ NEAA, no essential amino acids (g 100 g^−1^ dry weight).

**Table 3:** Sequences of primers corresponding to the reference and differential genes used in the RT-qPCR analysis.

Gene	GenBank ID	Forward Primer (5′-3′)	Reverse Primer (5′-3′)	Reference
Differential genes				
*Casp3*	DQ345773	CTGATTTGGATCCAGGCATT	CGGTCGTAGTGTTCCTCCAT	[31]
*Casp9*	DQ345775	GGCAGGACTCGACGAGATAG	CTCGCTCTGAGGAGCAAACT	[31]
*CD4*	AM491135	GTGATAACGCTGAAGATCGAGCC	GAGGTGTGTCATCTTCCGTTG	[39]
*CD8α*	AJ846849	CCACTGCATGCGCCGGTGAGG	GTGGACGTCGACTTAAACGTG	[39]
*COX2*	AJ630649	CATTCTTTGCCCAGCACTTCACC	AGCTTGCCATCCTTGAAGAGTC	[31]
*Fth*	FJ197144	AACCATGAGTTCTCAGGTGAG	TTAGCTGCTCTCTTTGCCCAG	[38]
*Ig*	AJ400233	GAGCTGCAGGAGGACAGTG	TCAGACTGGCCTCACAGCT	[31]
*IL-10*	AM268529	ACCCCGTTCGCTTGCCA	CATCTGGTGACATCACTC	[31,40]
*IL-1β*	AJ537421	ATTACCCACCACCCACTGAC	TCTCTTCCACTATGCTCTCCAG	[31]
*IL-6*	AM490062	ACTTCCAAAACATGCCCTGA	CCGCTGGTCAGTCTAAGGAG	[31]
*IL-8*	AM490063	GTCTGAGAAGCCTGGGAGTG	GCAATGGGAGTTAGCAGGAA	[31]
*Muc2*	Fasta	TCCATTTCCACCTACAACACAG	CATACTACAACGCAGGCGTA	[41]
*TCR β*	AJ493441	GACGGACGAAGCTGCCCA	TGGCAGCCTGTGTGATCTTCA	[31]
*TGF β*	AM421619	GACCTGGGATGGAAGTGG	CAGCTGCTCCACCTTGTG	[31]
*TNF α*	DQ200910	ACAGCGGATATGGACGGTG	GCCAAGCAAACAGCAGGAC	[31]
*Trf*	FJ197145	CAACAGTATGGGTGCTGACG	ACTGGCAGAGCACTTGGACT	[38]
Reference genes				
*EF1α*	AJ866727	GCTTCGAGGAAATCACCAAG	CAACCTTCCATCCCTTGAAC	[42]
*GADPH*	AY863148	GTGCCAGCCAGAACATCAT	TGTCGTCATATTTGGCGGGTTTC	[30]
*βact*	AJ269472	ATGTGGATCAGCAAGCAGG	AGAAATGTGTGGTGTGGTCG	[31]

**Table 4:** Growth and productive performance characteristics of European seabass fed different diets.

	30 FM	15 FM	0 FM	SEM	*p* Value
Initial weight (g)	40.45	40.29	38.25	0.93	n.s.
Final weight (g)	347.0 ^a^	341.38 ^a^	269.49 ^b^	8.70	0.0013
Weight gain (%)	757.85 ^a^	747.31 ^a^	604.55 ^b^	3.22	0.002
SGR (% day^−1^) ^1^	1.09 ^a^	1.09 ^a^	0.99 ^b^	0.02	0.0263
FI (g 100 g^−1^ fish day^−1^) ^2^	1.19	1.12	1.19	0.02	n.s.
FCR ^3^	1.47	1.52	1.58	0.05	n.s.
Survival (%)	93.33 ^a^	92.67 ^a^	84.00 ^b^	2.46	0.0465

Mean ± standard error (n = 3). Means with different subscripts in lowercase (a, b) indicate significant differences at *p* < 0.05, ^1^ SGR = 100 × ln (final weight/initial weight)/days. ^2^ FI (g 100 g^−1^ fish day^−1^) = 100 × feed consumption (g)/average biomass (g) × days. ^3^ FCR = feed consumption (g)/biomass gain (g). n.s.: no statistically significant differences (*p* > 0.05).

**Table 5:** Final biometric parameters of European seabass fed with the different experimental feeds.

	30 FM	15 FM	0 FM	SEM	*p* Value
CF (g cm^−3^) ^1^	1.34 ^b^	1.46 ^a^	1.48 ^a^	0.04	0.032
VSI (%) ^2^	11.82 ^b^	14.04 ^a^	13.41 ^ab^	0.50	0.049
HSI (%) ^3^	2.16 ^a^	1.91 ^ab^	1.83 ^b^	0.09	0.042
MF (%) ^4^	5.26 ^b^	7.30 ^a^	5.66 ^b^	0.50	0.048

Means of triplicate groups. Values are presented as mean ± SEM (standard error of the pooled means). Values in the same row with different superscript letters are significantly different (*p* < 0.05). ^1^ Condition factor CF = 100 × (total fish weight (g)/total length^3^ (cm)). ^2^ Viscerosomatic index (%) VSI = 100 × (visceral weight (g)/fish weight (g)). ^3^ Hepatosomatic index (%) HSI = 100 × (liver weight (g)/fish weight (g)). ^4^ Mesenteric fat (%) MF = 100 × (mesenteric fat weight (g)/fish weight (g)).

**Table 6:** Whole-body composition and retention efficiency of European seabass at the end of the experiment expressed in wet matter and retention efficiency indices.

	INITIAL	30 FM	15 FM	0 FM	SEM	*p* Value
Body Composition						
Dry matter (%)	26.62	36.91 ^b^	39.56 ^a^	37.77 ^ab^	0.63	0.047
Protein (%)	16.12	17.45	17.42	17.51	0.36	0.085
Fat (%)	4.66	15.86	18.30	16.76	0.76	0.072
Ash (%)	5.48	3.71	4.17	4.14	0.20	0.068
Energy (kJ g^−1^)	5.18	9.86	11.05	10.33	0.30	0.078
Retention efficiency						
PPV ^1^ (%)		25.34	23.95	24.12	0.87	0.082
PFV ^2^ (%)		76.35 ^ab^	84.25 ^a^	69.77 ^b^	3.81	0.040
PEV ^3^ (%)		29.24	32.10	29.82	1.18	0.096

Values are represented as mean ± standard error (n = 3) for each treatment. Superscripts with different letters indicate differences statistically significant at *p* < 0.05 by the Newman-Keuls test. ^1^ PPV (%) = Retained protein (final fish protein × Final biomass (g)) × 100 − Initial fish protein × initial biomass (g)/Protein ingested (kg of food ingested × % crude protein of feed). ^2^ PFV (%) = Retained Fat (final fish fat × Final biomass (g)) × 100 − Initial fish fat × initial biomass (g)/Fat ingested (kg food ingested × % crude fat of feed). ^3^ PEV (%) = Energy retained (Final fish energy × Final biomass (g)) × 100 − Initial fish energy × initial biomass (g)/Energy ingested (kg feed ingested × % feed energy).

**Table 7:** Amino acid retention efficiency in seabass at the end of the experiment.

	30 FM	15 FM	0 FM	SEM	*p* Value
EAA (%)					
Arginine	31.40 ^a^	25.66 ^ab^	19.44 ^b^	2.39	0.041
Histidine	23.16	27.83	31.42	3.36	0.210
Isoleucine	27.93	28.09	36.50	3.76	0.182
Leucine	22.31	25.49	28.08	2.79	0.069
Lysine	32.78	38.11	37.18	4.18	0.125
Methionine	18.20	20.22	21.23	1.04	0.069
Phenylalanine	18.38	20.45	19.95	2.22	0.063
Threonine	28.66 ^b^	37.28 ^ab^	46.96 ^a^	4.18	0.042
Valine	25.02	29.98	33.55	3.03	0.131
NEAA (%)					
Alanine	35.67 ^b^	48.58 ^b^	70.66 ^a^	5.36	0.045
Aspartic acid	31.57 ^b^	37.34 ^ab^	52.43 ^a^	4.80	0.050
Cysteine	20.37 ^a^	14.64 ^ab^	9.36 ^b^	2.41	0.049
Glutamic acid	14.79	15.80	18.76	1.38	0.072
Glycine	43.44 ^b^	56.20 ^ab^	72.39 ^a^	5.49	0.045
Proline	21.15	18.94	18.94	2.54	0.642
Serine	22.89	25.67	30.15	3.16	0.118
Tyrosine	20.98	23.63	24.55	3.19	0.187

Data are expressed as means ± SEM (n = 3). Means with different superscript letters in the same row significantly differ at *p* < 0.05. EAA: Essential Amino Acids. NEAA: Non-Essential Amino Acids.

**Table 8:** Relative expression of the genes analyzed in seabass.

Gene	30 FM	15 FM	0 FM	*p* Value
*Casp9*	0.025 ± 0.0030	0.026 ± 0.0030	0.022 ± 0.0028	n.s.
*TCR β*	0.004 ± 0.0022	0.007 ± 0.0022	0.007 ± 0.0021	n.s.
*IL-8*	0.085 ± 0.0580	0.112 ± 0.0580	0.215 ± 0.0547	n.s.
*IL-10*	0.326 ± 0.1101	0.298 ± 0.1101	0.230 ± 0.1038	n.s.
*TGF β*	0.009 ± 0.0023	0.013 ± 0.0023	0.001 ± 0.0022	n.s.
*TNF α*	0.078 ± 0.0214	0.026 ± 0.0214	0.027 ± 0.0202	n.s.
*Muc2*	10.273 ^a^ ± 0.1103	22.527 ^b^ ± 0.0745	8.458 ^a^ ±0.1103	<0.0001
*Fth*	0.015 ± 2.8715	0.043 ± 1.7029	0.006 ± 4.4524	n.s.
*βact*	0.002 ^a^ ± 0.0008	0.006 ^b^ ± 0.0008	0.001 ^a^ ± 0.007	0.0012

Data in the same row with different superscripts differ by a value of *p* < 0.05. SEM: standard error of the mean. n.s.: no statistically significant differences (*p* > 0.05).

**Table 9:** Economic analysis of seabass production fed different experimental diets.

	30 FM	15 FM	0 FM	SEM	*p* Value
Diet Price (€ kg^−1^)	1.12	1.21	1.31		
E.C.R. (€ kg^−1^) ^1^	1.65 ^c^	1.84 ^b^	2.07 ^a^	0.06	0.001
E.P.I. (€ fish^−1^) ^2^	1.34 ^a^	1.29 ^b^	0.97 ^c^	0.05	0.002

Means of triple groupings. Values are reported as mean ± SEM (standard error of the pooled means). A significant difference (*p* < 0.05) exists between values in the same row with different superscript letters. ^1^ E.C.R (Economic Conversion Ratio) = FCR × Diet Price. ^2^ E.P.I (Economic Profit Index) = [weight gain (kg) × selling price (€ kg^−1^)] − [weight gain (kg) × diet price (€ kg diet^−1^)]. (Considering a selling price of 5.5 €/kg for conventional seabass. The price of ingredients was: fishmeal (1400 €/ton), fish oil (2000 €/ton), wheat gluten (1750 €/ton), wheat (254 €/ton), soymeal (366 €/ton), soy oil (1139 €/ton), calcium phosphate (3500 €/ton), lysine (2000 €/ton), arginine (2000 €/ton), taurine and methionine (2000 €/ton), vitamin complex (5000 €/ton).

## Data Availability

The data presented in this study are available on request from the corresponding author.

## Supplementary Materials

The following supporting information can be downloaded at: https://www.mdpi.com/article/10.3390/ani16203172/s1, Figure S1: Illustrates the variability in the Ct values of the *β-Act* and *EF1α* genes, as depicted in a box and whiskers plot; Figure S2: Real-time quantitative polymerase chain reaction (RT-qPCR) for each differential gene using sample pools in European seabass fed experimental diets; Figure S3: Variation in the calibrators used for the differential and reference genes on different plates; Table S1: Variation in the calibrators used for the differential and reference genes on different plates.
